# Targeted phage aerosol for environmental control of CR-Kpn in the ICU: a prospective intervention study

**DOI:** 10.3389/fpubh.2026.1868215

**Published:** 2026-07-16

**Authors:** Yun Huang, Yanyu Huang, Ying Jin, Yuqin Xue, Yijing Zheng, Yun Gui, Zhizhuan Hou, Heping Dong, Qin Zhang, Minhua Xu, Xiaomin Zhao, Yating Wang, Yi Shi

**Affiliations:** 1 Department of Clinical Laboratory, Punan Branch of Renji Hospital, Shanghai Jiaotong University School of Medicine, Shanghai, China; 2 Department of Healthcare-Associated Infection Management, Punan Branch of Renji Hospital, Shanghai Jiaotong University School of Medicine, Shanghai, China; 3 Medical Intensive Care Unit, Punan Branch of Renji Hospital, Shanghai Jiaotong University School of Medicine, Shanghai, China; 4 Department of Central Laboratory, Punan Branch of Renji Hospital, Shanghai Jiaotong University School of Medicine, Shanghai, China

**Keywords:** environment, routine disinfection, ICU environment, phage aerosol, targeted carbapenem-resistant *Klebsiella pneumoniae* (CR-Kpn)

## Abstract

**Objective:**

This study aimed to evaluate the effect of targeted Carbapenem-resistant *Klebsiella pneumoniae* (CR-Kpn) phage aerosol combined with routine disinfection on the decontamination of CR-Kpn in the ICU environment, as well as its incidence rate, drug resistance rate, and antibiotic use.

**Methods:**

A prospective two-phase study design was adopted. During the baseline phase (P1), routine terminal disinfection with chlorine-containing agents was performed. In the intervention phase (P2), targeted phage aerosol spray was added to the isolation rooms of CR-Kpn patients. Multivariable models were fitted to isolate the intervention effect, adjusting for patient-days, imipenem use intensity, and carbapenem-resistant *Acinetobacter baumannii* (CRAB) incidence density. Outcomes including environmental CR-Kpn detection rate, incidence density, carbapenem resistance rate of *K. pneumoniae*, and antibiotic use intensity were compared between the two phases.

**Results:**

Following the combined intervention, the overall detection rate of CR-Kpn in the ICU environment decreased from 17.28% (47/272) to 0.96% (1/104), with the detection rate in sink drains dropping from 85.29% (29/34) to 7.69% (1/13). Clinical data showed that the incidence density of CR-Kpn significantly decreased from 10.16 cases/1,000 patient-days to 4.71 cases/1,000 patient-days (*p* = 0.007), whereas the incidence density of carbapenem-resistant *A. baumannii* (CRAB) increased significantly, suggesting the species-specificity of the intervention. Concurrently, the carbapenem resistance rate of *K. pneumoniae* decreased from 62.50 to 53.80%, and the use intensity of key antibiotics (tigecycline, polymyxin B, meropenem) significantly declined (reductions of 52.6, 58.5, and 36.6%, respectively; *p* < 0.01 for all).

**Conclusion:**

The combination of phage aerosol and routine disinfection showed potential in controlling CR-Kpn contamination in the ICU environment, reducing its incidence and carbapenem resistance rate, and decreasing the use of related antibiotics. This approach may provide a safe and effective biological disinfection strategy for controlling the spread of CR-Kpn.

## 1 Introduction

Carbapenem-resistant *Klebsiella pneumoniae* (CR-Kpn) is one of the “major clinical threat pathogens” frequently causing outbreaks in the intensive care units (ICUs). It spreads rapidly through close contact or contaminated surfaces and exhibits strong resistance to conventional disinfection methods (1). Despite intensive global efforts, the control of CR-Kpn in healthcare settings remains a major challenge. Antibiotic development against carbapenem-resistant Enterobacteriaceae has been slow, with only a few novel agents (e.g., ceftazidime-avibactam, meropenem-vaborbactam) reaching clinical use, and resistance to these has already emerged (2) Vaccine research against *K. pneumoniae* has made progress, including immunoinformatics-based multi-epitope vaccines (3), yet no licensed vaccine is available for CR-Kpn. Understanding microbial signatures and virulence factors (e.g., hypermucoviscosity, siderophores) has improved risk stratification, but these insights have not translated into environmental control measures (4, 5). Even phage therapy, despite its promise, is primarily investigated for treating established infections rather than for environmental decontamination. Thus, a critical knowledge gap remains: whether phage-based disinfection can be practically and safely integrated into ICU environmental hygiene to reduce CR-Kpn transmission. While these medical countermeasures are under development, environmental disinfection remains the most immediately deployable strategy to interrupt nosocomial transmission. However, current environmental disinfection strategies commonly used in ICUs, such as ultraviolet irradiation and chemical disinfectants, still have certain limitations. These include the gradual increase in pathogen tolerance to disinfectants, the difficulty in balancing effective concentration with the safety window of disinfectants (too high a concentration may leave toxic residues, while too low may be ineffective), and the frequent presence of blind spots during terminal disinfection in practice, making it challenging to thoroughly eliminate environmental pathogens in areas such as sink drains (6). Studies have reported that even after routine disinfection procedures, the contamination rate of CR-Kpn in the ICUs environment remains as high as 3.31% (7), this could be driving the steady rise in CR-Kpn isolation rates within ICUs (8). Therefore, exploring new disinfection strategies capable of effectively eliminating environmental CR-Kpn contamination is urgently significant for interrupting intra-hospital transmission chains and controlling related infections.

In recent years, bacteriophages have shown promising application prospects in the field of hospital infection control, owing to their specific bactericidal activity against multidrug-resistant bacteria, ability to eradicate biofilms, and favorable biosafety profile (9). A study by Wang et al. (7) first demonstrated that using phage-prepared aerosols for environmental intervention in the ICUs can significantly reduce the transmission risk of carbapenem-resistant *Acinetobacter baumannii* (CRAB), providing important evidence for the practical application of phages in hospital environmental disinfection (10). Nevertheless, evidence for phage aerosol against CR-Kpn is lacking. To date, no prospective study has evaluated the effectiveness of phage aerosol specifically targeting CR-Kpn in ICU environments, nor has any study systematically assessed its impact on clinical outcomes such as infection incidence, antimicrobial resistance rates, and antibiotic consumption. This constitutes the specific knowledge gap that our study aims to fill (11–13).

To address this gap, we conducted a prospective two-phase intervention study. First, we isolated and characterized a broad-spectrum lytic phage (Ph-kp8) against clinically prevalent CR-Kpn strains. We then implemented a combined disinfection protocol consisting of routine chlorine-based disinfection plus targeted phage aerosol in ICU isolation rooms. The primary objectives were: to assess the reduction in environmental CR-Kpn contamination, particularly in high-risk sites such as sink drains; to evaluate the impact on CR-Kpn incidence density, carbapenem resistance rate, and antimicrobial use density (AUD); and to confirm biosafety by monitoring phage residue. By comparing baseline (routine disinfection alone) and intervention phases, we sought to determine whether phage aerosol can serve as an effective and safe adjunctive environmental control measure against CR-Kpn in the ICU.

## 2 Materials and methods

### 2.1 CR-Kpn isolation and identification

From January 1, 2022, to December 31, 2024, active screening for CR-Kpn was performed on patients admitted to the ICU with a length of stay >48 h. A total of 209 CR-Kpn strains were collected. Inclusion criteria: first-time CR-Kpn detection (infection or colonization) in ICU patients with length of stay >48 h. Exclusion criteria: duplicate isolates from the same patient within 7 days, non-ICU patients, and rooms without environmental sampling. Strain identification was conducted using the VITEK-MS mass spectrometry system (bioMérieux, France). Antimicrobial susceptibility tests were performed using the VITEK-2 system (bioMérieux, France) as recommended by the Clinical and Laboratory Standards Institute (CLSI) M100, 32nd edition (14).

### 2.2 Bacteriophage isolation, genomic and bioinformatics analysis

**Figure 1:**
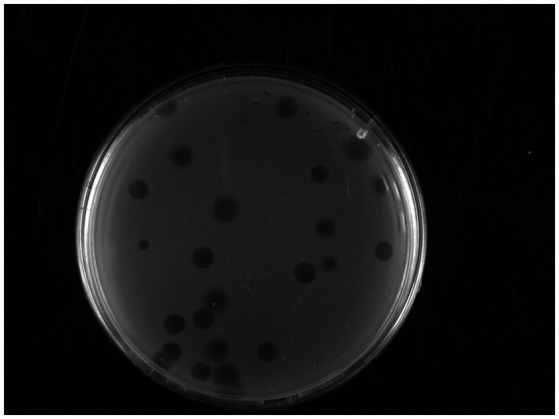
Plaques of phage Ph-kp8.

**Figure 2:**
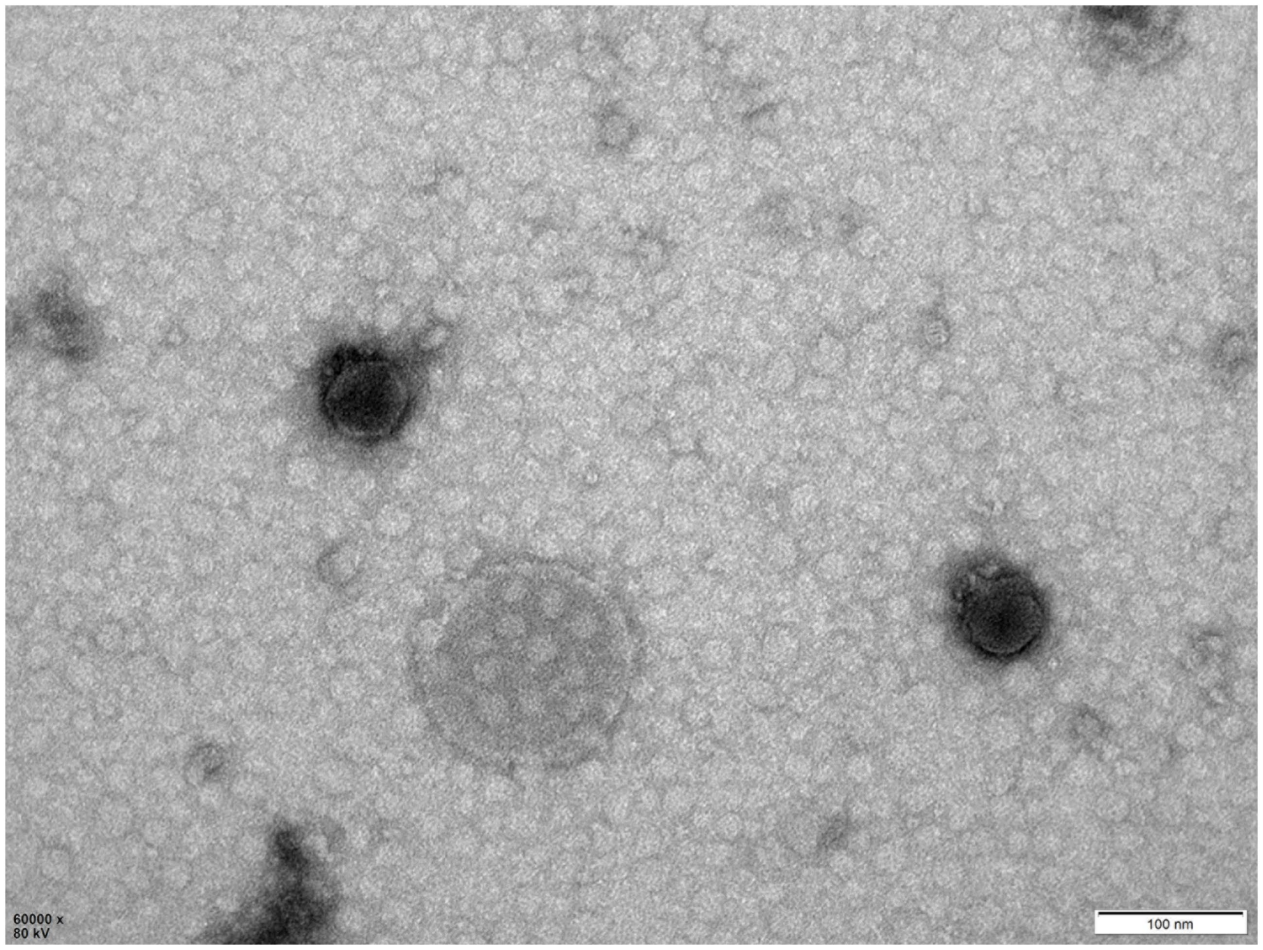
Transmission electron micrograph of phage Ph-kp8 (60,000×, 80 kV). The phage exhibits a regular hexagonal head with a diameter of 40–60 nm and a very short, nearly invisible tail, indicated by the arrow.

Bacteriophage Isolation and Screening: From 2022 to 2024, 18 anti-CR-Kpn phages were isolated from untreated sewage samples collected in our hospital. Host range identification confirmed one broad-spectrum phage (Ph-kp8), which could lyse 47.85% (100/209) of the clinical CR-Kpn strains (Figures 1, 2). This phage was therefore selected for subsequent experiments.

Whole-genome sequencing of the broad-spectrum phage (Ph-kp8) was performed, and its genomic characteristics and functional composition were analyzed using bioinformatics methods. The annotated genome sequence is available under GenBank accession number PZ466138.

### 2.3 CRAB identification and susceptibility testing

CRAB strains were identified from clinical specimens using the same VITEK-MS system as for CR-Kpn. Antimicrobial susceptibility to meropenem and imipenem was determined by the VITEK-2 system and interpreted according to CLSI M100 32nd edition criteria (15). CRAB was defined as *A. baumannii* isolates resistant to meropenem or imipenem (MIC ≥ 8 μg/mL). To assess the species specificity of the intervention, the lytic activity of Ph-kp8 against 20 randomly selected CRAB clinical isolates was tested by spot assay; no lytic activity was observed against any CRAB isolate.

### 2.4 Genomic analysis of CR-Kpn strains for phylogenetic relatedness

To address the potential for strain replacement and to confirm the clonality of the CR-Kpn population in our ICU, we conducted whole-genome sequencing on a subset of strains. A total of six CR-Kpn strains isolated from the ICU environment after disinfection during both the P1 and P2 phases, representative of the circulating populations, were selected for sequencing. Genomic DNA was sequenced on the DNBSEQ-T7 platform. Following quality control, reads were *de novo* assembled. A core-genome single nucleotide polymorphism (SNP) analysis was performed using Snippy v4.6.0 against the reference genome CP180580.1. A maximum-likelihood phylogenetic tree was then constructed based on the core SNP alignment using IQ-TREE v1.6.12 with 1,000 bootstrap replicates, to assess the genetic relatedness of the strains. CP180580.1 is a complete genome of a CR-Kpn strain (ST11, carrying blaKPC-2) isolated from a hospitalized patient in China, representing the dominant carbapenemase genotype and sequence type circulating in our region, which makes it a suitable reference for core-genome SNP analysis.

### 2.5 Study design

Baseline phase (P1): From January 1 to December 31, 2024, baseline data were collected in the ICU, including environmental CR-Kpn contamination before and after disinfection, CR-Kpn incidence density, the carbapenem resistance rate of *K. pneumoniae*, and antimicrobial use density.

Intervention Phase (P2): From January 1 to August 31, 2025 (8 months), targeted CR-Kpn phage aerosol spray was added to the routine disinfection protocol.

To assess the specificity of the intervention, data on CRAB detection were simultaneously collected as a control. The study was reviewed by the Ethics Committee of Punan Branch of Renji Hospital, Shanghai Jiaotong University School of Medicine, which confirmed that the project did not involve human participants or human-related ethical issues, and therefore formal ethical approval was not required. Data confidentiality was strictly protected, and all information was handled in accordance with institutional guidelines.

### 2.6 Environmental disinfection protocol

The study was conducted in two 20 m^2^ ICU isolation rooms.

P1 Phase (Routine Disinfection): routine terminal decontamination of the environment was performed using chlorine-containing disinfectant at a concentration of 500 mg/L for cleaning large surfaces (e.g., equipment panels, monitors, bed units), and sinks. An alcohol-based preparation (75%) for rapid disinfection of small surfaces was also used.

During the P2 phase (combined disinfection), after completing the routine disinfection procedures from the P1 phase, terminal disinfection was additionally performed by spraying a suspension of the broad-spectrum phage Ph-kp8. Prior to spraying, the lytic activity of the phage against current clinical CR-Kpn isolates was confirmed using the agar overlay method. A commercial ultrasonic humidifier (Model WH-U900, Zhongshan, China) was used for aerosolization. The device generated aerosol particles with a mass median aerodynamic diameter (MMAD) of 2.5–4.5 μm, as specified by the manufacturer and verified by laser diffraction particle sizing. The nebulization flow rate was approximately 3.0 mL/min, with an output velocity of 0.5 m/s at the nozzle exit. The humidifier was positioned at the center of the room at a height of 1.2 m. The phage suspension (1,500 mL at 1 × 10^10^–1 × 10^11^ PFU/mL) was aerosolized over approximately 8–10 min to ensure uniform coverage of the 20 m^2^ isolation room. These parameters were selected based on preliminary optimization experiments to maximize surface deposition while minimizing droplet settling time.

### 2.7 Environmental sampling protocol

Sampling time points and frequency

P1 Phase: Samples were collected within 1 h after routine chlorine-based disinfection.

P2 Phase: Samples were collected at two time points:

Within 1 h after combined disinfection to assess CR-Kpn residue.

At 1 h and 4 h after phage spraying to confirm no phage residue, ensuring subsequent patient safety.

Sampling sites

Eight high-touch, contamination-prone sites were sampled in each isolation room: patient bed rail, monitor screen/buttons, infusion pump buttons, stethoscope, isolation room door handle, sink basin, sink drain, and faucet.

Sampling method

Surface samples were collected according to GB15982-2012 “Hygienic Standard for Disinfection in Hospitals” (15). A swab was used to sample a 5 cm × 5 cm area on flat surfaces. The swab was placed in a tube with neutralizer for transport. It was then streaked onto blood agar plates, which were incubated at 35 °C for 48 h for analysis.

### 2.8 Post-intervention phage residue monitoring

To ensure biosafety and confirm the absence of residual aerosolized phage, environmental samples were routinely collected during the intervention phase (P2) from high-touch surfaces (bed rails, monitors, etc.) at 1 h and 4 h after phage spraying as part of the environmental monitoring protocol. Samples were eluted in SM buffer, filtered through 0.22 μm filters, and spotted onto double-layer agar plates seeded with the host CR-Kpn strain (Ph-kp8’s original host). After overnight incubation at 37 °C, the absence of plaque-forming units (PFUs) confirmed no detectable viable phage particles. Positive controls (samples spiked with a known phage titer) validated assay sensitivity. No phage activity was detected in any post-intervention sample. The detection limit of the plaque assay was approximately 10 PFU per sample, as determined by spiking experiments with known phage titers. All positive controls (samples spiked with 10^3^ PFU of Ph-kp8) showed confluent lysis, confirming assay sensitivity. Raw data for all samples are provided in Supplementary Table S1.

### 2.9 Outcome measures

Environmental CR-Kpn residue rate: Defined as the percentage of CR-Kpn-positive environmental samples, calculated as (number of positive samples / total samples) × 100%. Rates were determined before (baseline contamination) and after disinfection (residual contamination).

CR-Kpn incidence density: The number of new CR-Kpn cases (first-time detection of infection or colonization) per 1,000 patient-days among patients with an ICU stay >48 h. Repeat isolates from the same patient were excluded. Calculated as (new cases/total patient-days) × 1,000. Data on carbapenem-resistant *A. baumannii* (CRAB) were collected concurrently as a control to assess the specificity of the intervention.

Carbapenem resistance rate: The proportion of *K. pneumoniae* isolates resistant to carbapenems (meropenem or imipenem).

Antimicrobial use density (AUD): Defined as the number of defined daily doses (DDDs) of specific anti-CR-Kpn antibiotics (tigecycline, polymyxin B, meropenem, imipenem) per 100 patient-days, following WHO recommendations.

### 2.10 Statistical analysis

Data were analyzed using WHONET 5.6, SPSS 20.0, and R (v4.3.0). Continuous data are presented as mean ± standard deviation and compared using the *t*-test (or Mann–Whitney *U* test for non-normal distributions). Categorical data are expressed as numbers (percentages) and compared using the *χ*^2^ test or Fisher’s exact test. A two-sided *p* < 0.05 was considered statistically significant.

For a more rigorous assessment of the primary outcomes accounting for potential ICU-level confounders, the main conclusions were based on multivariable models fitted with monthly aggregated data. The primary outcomes were: (a) CR-Kpn incidence density, modeled using negative binomial regression with an offset for patient-days; and (b) tigecycline use intensity, modeled using linear regression. The negative binomial model was chosen because the variance of monthly CR-Kpn incidence density substantially exceeded its mean (overdispersion test: *p* < 0.01), violating the equidispersion assumption of Poisson regression. Both models adjusted for three key covariates representing major ICU-level factors: total patient-days (a proxy for patient turnover and case-mix), imipenem use intensity (a proxy for antimicrobial stewardship changes), and CRAB incidence density (a proxy for competitive pathogen pressure). The results are reported as adjusted incidence density ratios (from the negative binomial model) or adjusted mean differences (from the linear model).

## 3 Results

### 3.1 Whole-genome sequencing and bioinformatics analysis of the broad-spectrum phage Ph-kp8

**Figure 3:**
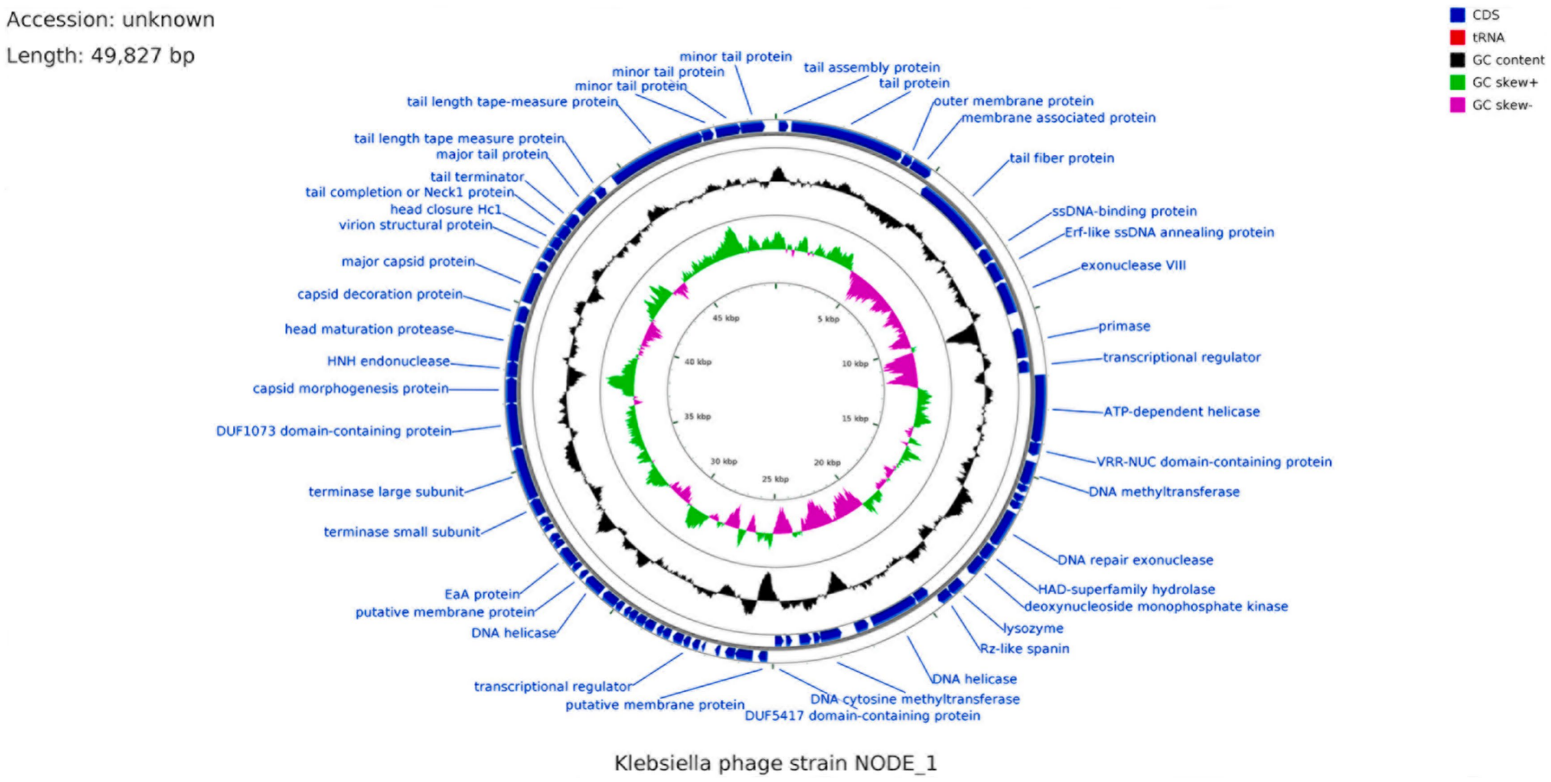
Genomic map of phage Ph-kp8. The outermost circle represents coding sequences (CDs) and structural RNA genes on the forward and reverse strands, with arrows indicating the direction of transcription (clockwise for the forward strand, counterclockwise for the reverse). The inner circles display regional GC content variation, followed by the GC skew (green for skew+, indicating an excess of G over C; purple for skew-, indicating an excess of C over G). The innermost circle shows the genomic coordinates (kb).

Whole-genome sequencing revealed that the broad-spectrum phage Ph-kp8 is a circular double-stranded DNA virus with a genome size of 49.8 kb, featuring a typical modular phage gene structure. Functional annotation indicated that its encoded proteins are primarily involved in key biological processes such as capsid assembly, DNA replication, and host lysis. Notably, no known virulence or antibiotic resistance genes were detected in the genome. Based on its biological characteristics, Ph-kp8 was identified as a virulent phage. These collective features demonstrate the favorable biosafety profile and potential therapeutic applicability of Ph-kp8, supporting its use in subsequent *in vitro* lysis assays and antimicrobial performance evaluations (Figure 3).

### 3.2 Genomic evidence for a clonal CR-Kpn population

Of the seven CR-Kpn isolates recovered from environmental samples after disinfection (one from P1 sink drain, one from P2 sink drain, and five from other sites across both phases; detailed in Table 1), six representative isolates (jinsuwen, heshenghua, zhouqichang, KPn-k83, kpn1, kpn2) were selected for whole-genome sequencing. The remaining isolate was not sequenced due to insufficient DNA yield.

**Table 1:** Comparison of CR-Kpn detection in environmental samples and disinfection efficacy between the intervention and baseline phase.

Sampling site	Phase	Samples (*n*)	CR-Kpn positive pre-disinfection n (%)	CR-Kpn positive post-disinfection n (%)	*p*-value (post- vs. pre-disinfection)	*p*-value (P2 vs. P1, post-disinfection)
Bed rail	P1	34	7 (20.59)	0 (0.00)	0.012	—
	P2	13	2 (15.38)	0 (0.00)	0.476	—
Monitor screen/buttons	P1	34	1 (2.94)	0 (0.00)	1	—
	P2	13	1 (7.69)	0 (0.00)	1	—
Infusion pump buttons	P1	34	1 (2.94)	0 (0.00)	1	—
	P2	13	0 (0.00)	0 (0.00)	—	—
Stethoscope	P1	34	0 (0.00)	0 (0.00)	—	—
	P2	13	1 (7.69)	0 (0.00)	1	—
Isolation room door handle	P1	34	0 (0.00)	0 (0.00)	—	—
	P2	13	1 (7.69)	0 (0.00)	1	—
Sink basin	P1	34	9 (26.47)	1 (2.94)	0.014	1
	P2	13	4 (30.77)	0 (0.00)	0.103	—
Sink drain	P1	34	29 (85.29)	5 (14.71)	<0.001	0.653
	P2	13	11 (84.62)	1 (7.69)	<0.001	—
Faucet	P1	34	0 (0.00)	0 (0.00)	—	—
	P2	13	1 (7.69)	0 (0.00)	1	—
Total	P1	272	47 (17.28)	6 (2.21)	<0.001	0.398
	P2	104	21 (20.19)	1 (0.96)	<0.001	—

P1 denotes the phase baseline phase, and P2 denotes the intervention phase.

“—” indicates that valid intergroup comparisons could not be performed due to zero positive samples after disinfection or limitations in sample structure.

Statistical comparisons were performed using Fisher’s exact test for 2 × 2 tables with expected cell counts <5, and the *χ*^2^ test for those with expected counts ≥5. *p*-values are presented without adjustment for multiple comparisons; given the exploratory nature of site-specific environmental analyses, these should be interpreted with caution.

**Figure 4:**
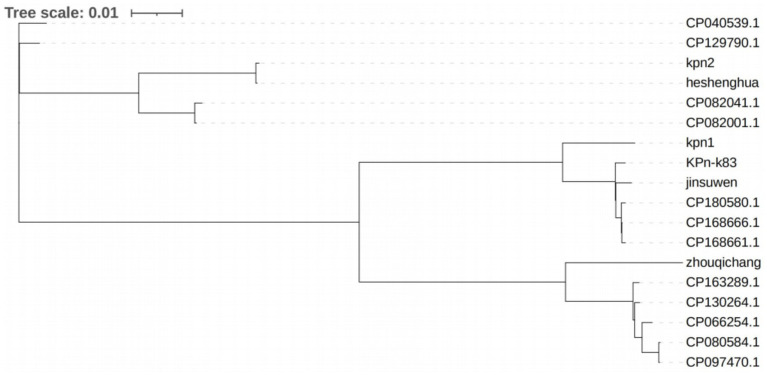
Phylogenetic tree of CR-Kpn strains based on core-genome SNPs. The scale bar represents a genetic distance of 0.01 substitutions per site. Isolates sequenced in this study (jinsuwen, heshenghua, zhouqichang, KPn-k83, kpn1, kpn2) are labeled with custom names; other tips are reference genomes from GenBank (including CP180580.1, used for read alignment). Bootstrap values are not shown.

Phylogenetic analysis based on core-genome SNPs showed that all six environmental isolates belonged to the same clonal lineage (sequence type 11 [ST11], as determined by in silico MLST) (Figure 4). Four isolates (jinsuwen, zhouqichang, KPn-k83, kpn1) formed a tight subcluster with fewer than 10 SNP differences, while the remaining two isolates (kpn2 and heshenghua) were slightly more divergent (30–50 SNP differences from the main cluster). This level of divergence is still within the range expected for ongoing transmission of a single lineage over several months, and all isolates shared the same carbapenemase gene (*bla*KPC-2) and exhibited identical mutations at key loci (e.g., a homozygous G → T transversion at position 218,592), supporting their common origin. The concurrent use of a virulent phage (Ph-kp8; DeePhage score: 0.72) against this clonal population provides strong genomic evidence that the intervention led to the reduction of the predominant resident strain, making widespread replacement by a genetically distinct clone unlikely.

### 3.3 Comparison of CR-Kpn detection before and after disinfection in P1 and P2 phases

Environmental sampling revealed comparable baseline contamination levels, with overall CR-Kpn detection rates of 17.28% (47/272) in P1 and 20.19% (21/104) in P2 prior to disinfection. Following disinfection, the overall detection rate was lower in the intervention phase (P2: 0.96%, 1/104) than in the baseline phase (P1: 2.21%, 6/272), although this difference was not statistically significant (*p* > 0.05).

Analysis of critical areas identified sink drains as the most heavily contaminated site, with pre-disinfection rates of 85.29% (29/34) in P1 and 84.62% (11/13) in P2. After disinfection, the CR-Kpn detection rate in sink drains was 7.69% (1/13) in P2, demonstrating a notable reduction compared to 14.71% (5/34) in P1, albeit without statistical significance. Furthermore, no CR-Kpn was detected on the inner sink surfaces post-disinfection in P2 (0/13), whereas a 2.94% (1/34) residual contamination rate was observed in P1. No CR-Kpn was detected on other high-touch surfaces—including bed rails, monitors, stethoscopes, door handles, and faucets—after disinfection in either phase (Table 1).

### 3.4 No detectable replication-competent phage residue post-intervention

As a critical biosafety assessment, we monitored for residual viable phage particles on environmental surfaces after aerosolization using culture assay. A total of 36 samples (collected from three isolation rooms at two time points) were tested. No lytic phage activity was detected in any post-intervention environmental samples collected from patient bed rails, monitors, or other high-touch surfaces. Positive controls (spiked samples with a known phage titer) confirmed assay sensitivity, indicating that the absence of detectable phage was not due to technical failure. These results collectively confirm that no replication-competent phages were detectable on high-touch surfaces at the time of patient admission (Supplementary Table S1).

### 3.5 Analysis of CR-Kpn incidence density, carbapenem resistance rate, and antimicrobial use intensity

In the P2 phase (January–August 2025), the combined disinfection intervention with targeted phage aerosol was implemented. During the study period, a total of 54 new CR-Kpn infection/colonization cases were identified in the ICU (40 cases in P1 over 3,938 patient-days; 14 cases in P2 over 2,971 patient-days). Consequently, the incidence density of CR-Kpn significantly decreased from 10.16 cases per 1,000 patient-days in the baseline phase (P1) to 4.71 cases per 1,000 patient-days in the intervention phase (P2) (*p* = 0.007).

In contrast, the epidemiological trend of CRAB during the same period showed an opposite pattern. New CRAB cases increased from 52 in P1 to 56 in P2, with the incidence density rising significantly from 13.21 to 18.85 cases per 1,000 patient-days (*p* = 0.048).

Carbapenem resistance monitoring also revealed divergent trends. The resistance rate of *K. pneumoniae* decreased from 62.50% in P1 to 53.80% in P2, while that of *A. baumannii* increased from 91.23 to 96.55%. Although both changes were not statistically significant (*p* > 0.05), the directional trends were opposite.

Compared with P1, most targeted antimicrobial AUD values in P2 showed significant reductions. Tigecycline AUD decreased from 24.05 to 11.41 DDDs/100 patient-days (52.6% reduction, *p* < 0.0001), polymyxin B from 1.35 to 0.56 (58.5% reduction, *p* = 0.0006), and meropenem from 20.54 to 13.02 (36.6% reduction, p < 0.0001). In contrast, imipenem use intensity increased significantly from 1.91 to 3.16 (65.4% increase, *p* = 0.0015), as shown in Table 2.

**Table 2:** Comparison of key antimicrobial use density (AUD) in the ICU between January–August 2024 and January–August 2025.

Antimicrobial agent	2024 AUD (DDDs/100 patient-days)	Jan-Aug 2025 AUD (DDDs/100 patient-days)	*p*-value
Tigecycline	24.05	11.41	<0.0001
Polymyxin B	1.35	0.56	0.0006
Meropenem	20.54	13.02	<0.0001
Imipenem	1.91	3.16	0.0015

AUD is expressed in DDDs per 100 patient-days.

**Figure 5:**
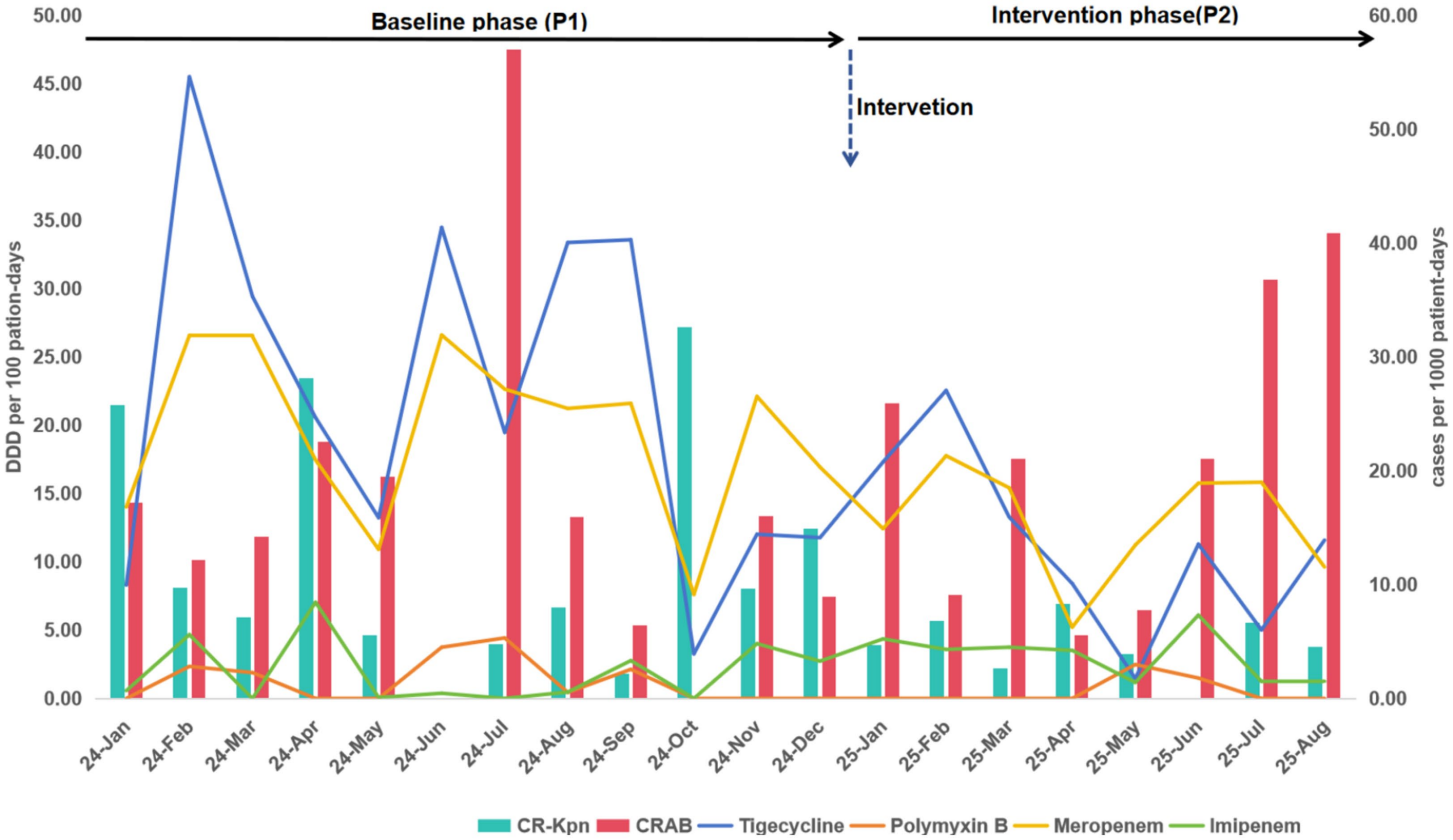
Temporal trends of CR-Kpn incidence density, CRAB incidence density, and key antimicrobial use intensity in the ICU during the baseline and intervention phases.

To control for potential temporal trends and ICU-level confounders, we further constructed multivariable-adjusted models. The negative binomial regression model revealed that the intervention phase (P2) remained independently associated with a significant reduction in CR-Kpn incidence density after adjusting for patient-days, imipenem use intensity, and CRAB incidence (adjusted incidence density ratio = 0.52, 95% CI: 0.35–0.78, *p* = 0.001). Similarly, the linear model demonstrated that the intervention phase was independently associated with a significant decrease in tigecycline use intensity after adjusting for the same covariates (adjusted mean difference = −10.5 DDDs/100 patient-days, *p* < 0.001), as shown in Table 3 and Figure 5

**Table 3:** Multivariable analysis of intervention effects on CR-Kpn incidence density and tigecycline use intensity.

Outcome variable	Predictor variable	Adjusted effect size (95% CI)	*p*-value
CR-Kpn incidence density	Intervention phase (P2 vs. P1)	0.52 (0.35–0.78)	0.001
	Patient-days (per 100-day increase)	1.02 (0.98–1.06)	0.32
	Imipenem AUD (per 1 DDD/100 patient-days)	1.03 (1.01–1.05)	0.005
	CRAB incidence (per 1 case/1000 patient-days)	1.05 (1.02–1.08)	0.001
Tigecycline use intensity	Intervention phase (P2 vs. P1)	−10.5 (−12.5 to −8.5)	<0.001
	Patient-days (per 100-day increase)	0.1 (−0.05 to 0.25)	0.19
	Imipenem AUD (per 1 DDD/100 patient-days)	0.2 (0.1–0.3)	0.001
	CRAB incidence (per 1 case/1000 patient-days)	0.5 (0.3–0.7)	<0.001

CR-Kpn incidence density results are expressed as incidence density ratios from negative binomial regression; tigecycline use intensity results are expressed as mean differences in DDDs/100 patient-days from linear regression. All models were adjusted for the listed ICU-level confounders. AUD, antimicrobial use density; CI, confidence interval.

## 4 Discussion

### 4.1 Summary of principal findings

In this prospective intervention study, we combined targeted CR-Kpn phage aerosols with conventional chlorine-based disinfection to systematically evaluate their effects on CR-Kpn contamination in the ICU environment, the incidence density of CR-Kpn in patients, the carbapenem resistance rate, and the intensity of key antibacterial drug use. The results showed that after the combined intervention, the overall detection rate of CR-Kpn in the environment decreased from 17.28% (47/272) to 0.96% (1/104). Although this environmental reduction did not reach statistical significance (*p* = 0.398, Table 1)—likely due to the limited sample size in the intervention phase (*n* = 104 vs. 272 in P1) and the already low post-disinfection positivity rates in both phases—the consistent directional trend across multiple sites supports a potential decontamination effect. Notably, the detection rate at the most challenging reservoir, the sink drains, decreased from 85.29% (29/34) to 7.69% (1/13), representing a clinically meaningful relative reduction of 58.5%, albeit not statistically significant. Clinically, the incidence density of CR-Kpn decreased significantly from 10.16 cases per 1,000 patient-days to 4.71 cases per 1,000 patient-days (*p* = 0.007), with a reduction of 53.6%. After adjusting for patient days, imipenem use intensity, and CRAB incidence density, the multivariable negative binomial regression model still showed that the intervention was independently associated with a decrease in incidence density (adjusted IRR = 0.52, 95% CI: 0.35–0.78, *p* = 0.001). At the same time, the use intensity of tigecycline, polymyxin B, and meropenem decreased by 52.6, 58.5, and 36.6%, respectively, (all *p* < 0.01). In terms of biological safety monitoring, at 1 h and 4 h after the intervention, no replication-competent phages were detected on all high-touch surfaces. Notably, the incidence density of CRAB, as a specific control, significantly increased during the same period (from 13.21 to 18.85 cases per 1,000 patient-days, *p* = 0.048), which not only confirmed the targeting effect of the intervention on CR-Kpn but also revealed the microecological changes caused by single-pathogen intervention. These findings suggest that phage aerosols, as an auxiliary disinfection method, may complement the deficiencies of conventional chemical disinfection in the ICU environment, showing favorable trends in challenging reservoirs such as sink drains, and exhibit good short-term biological safety.

### 4.2 The urgent need to improve existing disinfection strategies against CR-Kpn

The continuous contamination of CR-Kpn in the ICU environment, especially in the micro-environment protected by biofilms at sink drains, is a pressing problem that needs to be addressed urgently. Even if conventional chlorine-based disinfection is strictly carried out, it still leaves a large number of CR-Kpn in a viable state, becoming the source of continuous transmission. In the face of the slow development of new antibiotic research pipelines and the lack of licensed CR-Kpn vaccines, strengthening environmental disinfection is undoubtedly the most directly actionable intervention method to prevent the spread within the hospital. The limitations of existing methods—the tolerance of disinfectants, the toxicity of chemical residues, and the blind areas of disinfection—urgently require innovative auxiliary strategies, and these strategies must be both effective and safe. This study shows that phage aerosols provide a promising solution: they can specifically target CR-Kpn, leaving no toxic residues and causing little interference to the microbial ecology other than the target pathogen. Therefore, incorporating phage-based biological control into the routine disinfection protocol of the ICU should not merely be regarded as an option, but as a necessary evolution in the fight against carbapenem-resistant pathogens.

### 4.3 Comparison with previous phage-based disinfection studies

This study further enriches the evidence base for using phage aerosols as a tool for controlling biological contamination in hospital environments, and for the first time extends this strategy from CRAB to CR-Kpn. Chen et al. (16) published a pioneering study in 2022, where they used pre-optimized phage cocktail aerosols to significantly reduce environmental contamination and the incidence of patient infections in the ICU. This provided important empirical evidence for the application of phages in hospital disinfection. Subsequently, Tseng et al. (12) conducted a 4-year prospective study in the ECMO ward, adjusting the components of the phage cocktail through a spatial–temporal design, successfully reducing the hospital infection rate related to CRAB. However, all these studies focused on *A. baumannii*, while CR-Kpn differs significantly from CRAB (Carbapenem-resistant *A. baumannii*) in terms of drug resistance mechanisms, environmental survival capabilities, and biofilm formation characteristics. Therefore, they cannot be simply extrapolated. For instance, CR-Kpn is more likely to persist in biofilms in drainage pipes and gradually becomes more tolerant to chlorine-based disinfectants. To our knowledge, this study is the first prospective study in the ICU environment specifically designed and systematically evaluated the efficacy of phage aerosols for CR-Kpn. Compared with the study by Chen et al. (16), we not only focused on the reduction of environmental contamination rates, but also quantified the chain effects on patient morbidity density, changes in carbapenem resistance rates, and antibiotic consumption, and verified the specificity of the intervention through parallel monitoring of CRAB. Additionally, we used whole-genome sequencing to confirm that the prevalent CR-Kpn strains in the ICU were clonal populations, thereby eliminating the interference of “strain replacement” in the interpretation of the results. These methodological improvements enabled this study to more reliably link the environmental decontamination effect with clinical outcome improvements.

### 4.4 Environmental decontamination and the challenge of sink drains

The sink drain is widely recognized as one of the most important environmental reservoirs for multi-drug resistant bacteria in the ICU (17, 18). The reasons for this are as follows: The inner wall of the pipe is prone to form a dense biofilm, and the extracellular polymeric substances (EPS) in the biofilm not only hinder the penetration of disinfectants but also provide physical protection and a nutrient microenvironment for bacteria; in addition, the water flow from daily use can cause the CR-Kpn in the biofilm to detach and be aerosolized, forming bacterial aerosols with particle diameters typically less than 5 μm (19, 20). These aerosol particles (usually suspended in the air for several hours) can spread throughout the ICU along with the air flow from the air conditioning system, thereby initiating new colonization or even outbreak of infection in susceptible patients (4, 21). The data from the baseline period (P1) of this study once again confirmed this view: After conventional chlorine disinfection (500 mg/L), the detection rate of CR-Kpn in the sink drain remained as high as 14.71% (5/34), while other frequently touched surfaces (bed rails, monitors, stethoscopes, etc.) had all turned negative. This fully demonstrates that conventional chemical disinfection is difficult to completely remove the bacteria within the biofilm of the sink drain (22, 23).

What is more noteworthy is that even after the combined intervention, there was still one case of CR-Kpn detected at the sink drain. Possible explanations include: The spatial structure of the biofilm prevented the phage particles from penetrating deep into the biofilm, resulting in some bacteria not being lysed (24); The complex geometry of the drainage pipe (such as bends, threaded interfaces) caused uneven coverage of the phage aerosol (25); There may be subpopulations of bacteria that are insensitive to or have decreased sensitivity to Ph-kp8 (although our phages can lyse 47.85% of clinical isolates, more than half of the strains are insensitive) (25). To address these issues, future improvement strategies could include: using compound preparations of phages and biofilm degrading enzymes (such as polysaccharide depolymerase, DNase) or surfactants to enhance the penetration ability into mature biofilms; designing “cocktail” preparations containing multiple phages to expand the host range and delay the emergence of resistance; improving the aerosol generation device to increase the uniformity of coverage in concealed areas such as the interior of the pipes (26, 27). Additionally, regular replacement or physical clearing of the sink drains may also be an effective auxiliary measure to reduce the biofilm load (3, 28, 29).

### 4.5 Clinical benefits and antimicrobial stewardship implications

This study found that the effective control of environmental CR-Kpn contamination was highly consistent in time with the decrease in the incidence density of CR-Kpn in patients, and the association remained significant after multivariate correction. This association has a reasonable biological explanation: reducing the load of CR-Kpn in the environment, especially blocking the continuous dissemination from the aerosols in the drainage pipes, can reduce the colonization pressure on susceptible patients, thereby reducing subsequent infection events. It is notable that the decrease in the incidence density of CR-Kpn was not accompanied by a decrease in CRAB, but rather CRAB showed a significant increase. This precisely supports the species specificity of the intervention measures. If the decrease in CR-Kpn was due to strengthened overall infection control measures or a time trend, then CRAB should also show a similar trend, but the opposite was observed. Therefore, the parallel monitoring results of CRAB significantly enhanced the strength of causal inference.

In terms of the use of antibacterial drugs, the usage intensities of tigecycline, polymyxin B and meropenem all decreased significantly, with reductions of 52.6, 58.5 and 36.6%, respectively. These drugs are all “last line” drugs for treating CR-Kpn infections. The decrease in their consumption can bring benefits from two aspects: first, it reduces patients’ drug exposure and related adverse reactions (such as the nephrotoxicity of polymyxin B and the gastrointestinal reactions of tigecycline); second, it alleviates the selection pressure of broad-spectrum antibiotics on the intestinal flora and other bacteria in the ICU, which may delay the emergence of new resistance mechanisms. However, we also observed that the usage intensity of imipenem increased by 65.4%. This “see-saw effect” within the carbapenem class of drugs may reflect the compensatory behavior of clinicians after the stricter control of meropenem usage. This phenomenon warns us that antibacterial drug management (AMS) should not only focus on a single drug, but adopt a comprehensive strategy for the entire drug category. Otherwise, it may only be “picking up the pieces after knocking over a pot.” Future AMS interventions should simultaneously monitor the usage trends of all carbapenem drugs and make real-time adjustments based on the dynamic changes in the drug resistance spectrum of pathogenic bacteria.

### 4.6 Could phage aerosols partially replace chemical disinfectants?

Current environmental disinfection in the ICU is almost entirely dependent on chemical disinfectants (such as chlorine preparations, hydrogen peroxide, quaternary ammonium salts, etc.) and physical methods (such as ultraviolet rays, dry heat, etc.). However, chemical disinfectants have significant drawbacks that cannot be ignored: long-term use can induce bacteria to develop tolerance to the disinfectants (such as the upregulation of efflux pumps), and the chemical substances remaining in the environment may cause irritation to healthcare workers and patients, and even enter the environmental water bodies through the drainage system, causing ecological toxicity. Phage aerosols, as a biological disinfectant, have the following advantages: high specificity (only lysing the target bacteria, without harming the normal human flora and environmental microorganisms), biodegradability (no persistent chemical residues), and the ability to lyse bacteria in biofilms (some phages carry polysaccharide depolymerases). The safety monitoring of this study indicates that 1–4 h after the aerosol spraying, no replication-competent phage particles were detected on all sampling surfaces, which provides a safety guarantee for patients’ short-term admission.

Based on these observations, one might speculate whether phage aerosols could eventually complement or partially replace chemical disinfectants for certain low-risk surfaces in the ICU, such as bed rails or monitor shells. However, we emphasize that this remains a speculative, long-term vision rather than a conclusion supported by the current data. Several practical barriers must first be overcome: the compatibility of phages with chemical disinfectants (some formulations can inactivate phages), the cost of large-scale production and cold-chain logistics, the need for periodic reformulation to match circulating strains, and the potential for widespread phage resistance with chronic use. The present study was not designed to evaluate replacement strategies, and we caution against overinterpreting our findings in this direction. Future research specifically designed to compare chemical versus phage-based disinfection—ideally with randomized controlled designs and cost-effectiveness analyses—would be required before such a paradigm shift could be considered.

The choice of the 4-h time point for post-intervention phage residue monitoring warrants further explanation. This time point was selected because it represents the typical midpoint of the turnaround window (2–6 h) between terminal disinfection and next patient admission in our ICU. Aerosolized phage particles in the size range of 2.5–4.5 μm MMAD are expected to settle within 1–2 h under standard room ventilation (6–8 air changes per hour), with residual viability further diminished by desiccation and surface exposure. Importantly, our 1-h post-intervention samples also showed no detectable replication-competent phages on all high-touch surfaces (Supplementary Table S1). Thus, even in the uncommon scenario where a patient is admitted within 1–2 h of the procedure, our data suggest that surface contact would not pose a detectable phage exposure risk. We acknowledge that airborne phage particles were not directly sampled; however, patients are not present in the room during active aerosolization, and the rapid settling dynamics of the generated particles make surface contact the primary potential exposure route, which our sampling directly assessed. While we cannot formally exclude the presence of non-culturable phage particles or those below the detection limit, the absence of detectable replication-competent phages at 1 h post-intervention provides reasonable assurance of short-term safety for patients admitted soon after the procedure.

### 4.7 Study limitations

Several limitations should be considered when interpreting these findings. First, this was a single-center, pre-post intervention study rather than a randomized controlled trial. Although we adjusted for major time-dependent confounders (patient-days, imipenem use, and CRAB incidence), unmeasured variables such as nurse-to-patient ratios, seasonal variations in hand hygiene compliance, or changes in infection control staffing could have influenced outcomes. The intervention phase (P2) lasted only 8 months, and environmental sampling was less intensive (104 vs. 272 samples), particularly for sink drains (n = 13), which limited statistical power for site-specific comparisons. Second, we used a single phage (Ph-kp8) that lyses 47.85% of clinical CR-Kpn isolates, leaving more than half of potential strains insensitive. We did not monitor phage susceptibility among environmental strains during the intervention, so we cannot exclude the possibility that resistant subpopulations were selected. Third, while we confirmed the absence of detectable phage residues on surfaces by qPCR and culture, we did not sample the interior of drain biofilms, which could harbor phages for longer periods. Fourth, the significant rise in CRAB incidence density—a striking contrast to the decline in CR-Kpn—warrants attention. This might reflect ecological niche release (CRAB expanding after CR-Kpn suppression) or unrelated temporal trends; regardless, it highlights the potential for unintended microecological consequences of single-pathogen interventions. Finally, no formal cost-effectiveness analysis was performed. Scaling phage aerosol for routine use would require large-scale production, cold-chain logistics, periodic reformulation to match circulating strains, and specialized nebulization equipment, all of which add operational costs. Moreover, strain diversity across hospitals and regions limits the generalizability of Ph-kp8. Future multi-center, randomized trials with longer follow-up, metagenomic monitoring, phage cocktails, and regional phage libraries are needed to fully assess clinical value, ecological safety, and feasibility.

It is also important to acknowledge that the post-disinfection environmental CR-Kpn detection rate did not differ significantly between P1 and P2 (2.21% vs. 0.96%, *p* = 0.398, Table 1), partly due to the limited sample size in P2 (*n* = 104 vs. 272 in P1) and the low baseline positivity. Therefore, while the phage intervention showed a promising trend, the environmental data alone do not provide strong statistical evidence of superiority over routine disinfection. The significant reduction in CR-Kpn incidence density (10.16 to 4.71 per 1,000 patient-days, *p* = 0.007) was robust to multivariable adjustment, but as with any pre-post study, we cannot completely exclude the influence of unmeasured secular trends, such as changes in infection control staffing, hand hygiene compliance, seasonal variations in CR-Kpn prevalence, or other concurrent infection prevention interventions. The concurrent rise in CRAB incidence argues against a general improvement in infection control, but does not rule out pathogen-specific confounding. Future randomized controlled trials are needed to confirm causality.

## 5 Conclusion

In conclusion, this study is the first to demonstrate in a prospective, controlled design ICU environment that the targeted CR-Kpn phage aerosol combined with conventional chlorine-based disinfection can significantly reduce environmental CR-Kpn contamination (especially providing additional improvement for the stubborn reservoir of the sink drain), decrease the incidence density of CR-Kpn, and reduce the intensity of antibiotic use in the critical “last line of defense.” The safety monitoring detected no replication-competent phages on environmental surfaces, preliminarily supporting the safety of its near-term application. Despite the above limitations, this study provides new key evidence for the application of phages in hospital environmental disinfection and offers a potential, operational auxiliary strategy for addressing the persistent transmission of CR-Kpn in ICUs. In the future, with the development of phage cocktail design, optimization of aerosol devices, and rapid strain matching technologies, phage aerosols are expected to gradually move from “auxiliary measures” to “routine measures,” and may form a complement with chemical disinfectants to jointly build a more sustainable hospital environmental infection control system.

## Data Availability

The original contributions presented in the study are included in the article/Supplementary material, further inquiries can be directed to the corresponding author.

## References

[1] Benedict KL Brady HW Newsome AL. Viral disinfection of porous fomites utilizing a bacteriophage model and chlorine dioxide gas. Health Secur. (2023) 21:303–9. doi: 10.1089/hs.2022.0138, 37289796

[2] Rutala WA Weber DJ. Disinfection and sterilization in health care facilities: an overview and current issues. Infect Dis Clin N Am. (2021) 35:575–607. doi: 10.1016/j.idc.2021.04.004, 34362535

[3] Ahmad TA Tawfik DM Ghoneim HM Nabil MA El-Ashry EH El-Sayed LH. Variable progressive behavior of *Klebsiella pneumoniae* at different sites of infection. Front Immunol. (2026) 17:1775450. doi: 10.3389/fimmu.2026.1775450, 42051528 PMC13111035

[4] Lu YH Wu H Zhang HH Li WS Lai ACK. Synergistic disinfection of aerosolized bacteria and bacteriophage by far-UVC (222-nm) and negative air ions. J Hazard Mater. (2023) 441:129876. doi: 10.1016/j.jhazmat.2022.129876, 36087531

[5] Yan Z Zhou Y Du M Bai Y Liu B Gong M Prospective investigation of carbapenem-resistant *Klebsiella pneumonia* transmission among the staff, environment and patients in five major intensive care units, Beijing. J Hosp Infect (2019);101:150–157, doi: 10.1016/j.jhin.2018.11.019 30529506

[6] Beltran EO Allison JR Jakubovics NS Castellanos JE Holliday R Velandia-Romero ML . Control of viral aerosol dispersion during simulated dental procedures. Int Dent J. (2025) 75:103963. doi: 10.1016/j.identj.2025.103963, 41135414 PMC12593617

[7] Wang L Dong Q Tang K Han K Bai H Yin Y . Effect of phage spray on hatchability and chick quality of eggs contaminated with *Salmonella Typhimurium*. Viruses. (2024) 16:1338. doi: 10.3390/v16081338, 39205312 PMC11359902

[8] Di Bella S Sanson G Monticelli J Zerbato V Principe L Giuffrè M . *Clostridioides difficile* infection: history, epidemiology, risk factors, prevention, clinical manifestations, treatment, and future options. Clin Microbiol Rev. (2024) 37:e0013523. doi: 10.1128/cmr.00135-23, 38421181 PMC11324037

[9] Henriques TM Rito B Proenca DN Morais PV. Application of an ultrasonic nebulizer closet in the disinfection of textiles and footwear. Int J Environ Res Public Health. (2022) 19:10472. doi: 10.3390/ijerph191710472, 36078188 PMC9518335

[10] Skliros D Papazoglou P Gkizi D Paraskevopoulou E Katharios P Goumas DE . In planta interactions of a novel bacteriophage against *Pseudomonas syringae* pv. Tomato. Appl Microbiol Biotechnol. (2023) 107:3801–15. doi: 10.1007/s00253-023-12493-5, 37074382 PMC10175458

[11] van der Vossen J Kreikamp AP Hatt V Ouwens AMT Brasem DJ Heerikhuisen M . Establishment and application of test methodology demonstrating the functionality of air purification systems in reducing virus-loaded aerosol in indoor air. J Hosp Infect. (2023) 135:74–80. doi: 10.1016/j.jhin.2023.03.004, 36918067 PMC10008183

[12] Tseng CC Chen LK Chu HT Chen YT Jiang HL Yang HH . Prophylactic phage aerosols for nosocomial infection control in an extracorporeal membrane oxygenation unit: a 4-year prospective study of temporospatially designed phage cocktails. Int J Antimicrob Agents. (2025) 65:107413. doi: 10.1016/j.ijantimicag.2024.107413, 39709129

[13] Alian M Abdulla H Harb N. Phage-based biocontrol of multidrug-resistant *Staphylococcus aureus* and *Escherichia coli* in foods and on food-contact surfaces: toward sustainable food safety. J Appl Microbiol. (2026) 137:lxag022. doi: 10.1093/jambio/lxag022, 41562958

[14] Franke G Knobling B Brill FH Becker B Klupp EM Belmar Campos C . An automated room disinfection system using ozone is highly active against surrogates for SARS-CoV-2. J Hosp Infect. (2021) 112:108–13. doi: 10.1016/j.jhin.2021.04.007, 33864891 PMC8046700

[15] Schuetz AN Ferrell A Hindler JA Humphries R Bobenchik AM. Overview of changes in the clinical and laboratory standards institute performance standards for antimicrobial susceptibility testing: M100 32nd and 33rd editions. J Clin Microbiol. (2025) 63:e0162323. doi: 10.1128/jcm.01623-23, 40772786 PMC12421849

[16] Chen LK Chang JC Chu HT Chen YT Jiang HL Wang LS . Preoptimized phage cocktail for use in aerosols against nosocomial transmission of carbapenem-resistant *Acinetobacter baumannii*: a 3-year prospective intervention study. Ecotoxicol Environ Saf. (2022) 236:113476. doi: 10.1016/j.ecoenv.2022.113476, 35367880

[17] Sabri M El Handi K El Tousy A De Stradis A Elbeaino T. Synergistic antibacterial activity of Lactococcus lactis and Xylella phage MATE 2 for an effective biocontrol strategy against black rot disease in broccoli. Front Microbiol. (2024) 15:1468792. doi: 10.3389/fmicb.2024.1468792, 39224218 PMC11366581

[18] Vega II Rodriguez NER Perez VJA Velazquez APZ Sanchez VV Arana JLR . Bacteriophages as a biocontrol strategy to prevent the contamination of meat products with *Escherichia coli* - a meta-analysis. Pol J Microbiol. (2025) 74:165–76. doi: 10.33073/pjm-2025-014, 40544515 PMC12182924

[19] Shopova E Brankova L Ivanov S Urshev Z Dimitrova L Dimitrova M . *Xanthomonas euvesicatoria*-specific bacteriophage BsXeu269p/3 reduces the spread of bacterial spot disease in pepper plants. Plants (Basel). (2023) 12:3348. doi: 10.3390/plants12193348, 37836088 PMC10574073

[20] Liu Y Liu M Hu R Bai J He X Jin Y. Isolation of the novel phage PHB09 and its potential use against the plant pathogen *Pseudomonas syringae* pv. Actinidiae. Viruses. (2021) 13:2275. doi: 10.3390/v13112275, 34835081 PMC8622976

[21] Heinz E Brindle R Morgan-McCalla A Peters K Thomson NR. Caribbean multi-centre study of *Klebsiella pneumoniae*: whole-genome sequencing, antimicrobial resistance and virulence factors. Microb Genom. (2019) 5:e000266. doi: 10.1099/mgen.0.000266, 31038449 PMC6562249

[22] Kaple CE Memic S Cadnum JL Donskey CJ. Evaluation of an automated far ultraviolet-C light technology for decontamination of surfaces and aerosolized viruses in bathrooms. Antimicrob Resist Infect Control. (2024) 13:114. doi: 10.1186/s13756-024-01473-7, 39343973 PMC11441258

[23] Lu YH Wang RX Liu HL Lai ACK. Evaluating the performance of UV disinfection across the 222-365 nm Spectrum against aerosolized Bacteria and viruses. Environ Sci Technol. (2024) 58:6868–77. doi: 10.1021/acs.est.3c08675, 38593035

[24] Shi Y Zhang W Li L Wu W Li M Xiao K . Evaluation of phage-based decontamination in respiratory intensive care unit environments using ddPCR and 16S rRNA targeted sequencing techniques. Front Cell Infect Microbiol. (2024) 14:1442062. doi: 10.3389/fcimb.2024.1442062, 39224703 PMC11366697

[25] Kramer B Warschat D Meepool A Muranyi P. How to validate UV-C based air cleaners using viruses containing aerosols in a test room. J Appl Microbiol. (2024) 135:lxae287. doi: 10.1093/jambio/lxae287, 39544125

[26] Vila M Balcao LMN Balcao VM. Phage delivery strategies for biocontrolling human, animal, and plant bacterial infections: state of the art. Pharmaceutics. (2024) 16:374. doi: 10.3390/pharmaceutics16030374, 38543268 PMC10976114

[27] Zhang L Guo Y Tie J Yao Z Feng Z Wu Q . Grating-like DBD plasma for air disinfection: dose and dose-response characteristics. J Hazard Mater. (2023) 447:130780. doi: 10.1016/j.jhazmat.2023.130780, 36669408

[28] Liu F Ma Q Zhang J Wang J Govindan D Zhao M . Self-cleaning microwave-responsive MXene-coated filtration system for enhanced airborne virus disinfection. ACS Appl Mater Interfaces. (2025) 17:27167–77. doi: 10.1021/acsami.5c02969, 40273420

[29] Figueiredo CM Malvezzi Karwowski MS da Silva Ramos RCP de Oliveira NS Pena LC Carneiro E . Bacteriophages as tools for biofilm biocontrol in different fields. Biofouling. (2021) 37:689–709. doi: 10.1080/08927014.2021.1955866, 34304662

