# Supplementary material for: Targeted phage aerosol for environmental control of CR-Kpn in the ICU: a prospective intervention study
Source: Front Public Health. 2026 Jul 16;14:1868215. doi: 10.3389/fpubh.2026.1868215 (PMC13422463; doi:10.3389/fpubh.2026.1868215)
Supplement: Supplementary file 1 [file Table_1.docx]

**Supplementary Table S1**. Raw data of post-intervention phage residue detection by plaque assay

| Sample ID | Collection Date | Room No. | Time Post-Spray | Surface Type | PFU Count | Positive Control (spiked sample) |
| --- | --- | --- | --- | --- | --- | --- |
| PR-001 | 2025-03-10 | Room 101 | 1 h | Bed rail | 0 | Confluent lysis |
| PR-002 | 2025-03-10 | Room 101 | 1 h | Monitor | 0 | Confluent lysis |
| PR-003 | 2025-03-10 | Room 101 | 1 h | Door handle | 0 | Confluent lysis |
| PR-004 | 2025-03-10 | Room 101 | 4 h | Bed rail | 0 | Confluent lysis |
| PR-005 | 2025-03-10 | Room 101 | 4 h | Monitor | 0 | Confluent lysis |
| PR-006 | 2025-03-10 | Room 101 | 4 h | Door handle | 0 | Confluent lysis |
| PR-007 | 2025-03-18 | Room 102 | 1 h | Bed rail | 0 | Confluent lysis |
| PR-008 | 2025-03-18 | Room 102 | 1 h | Monitor | 0 | Confluent lysis |
| PR-009 | 2025-03-18 | Room 102 | 1 h | Infusion pump | 0 | Confluent lysis |
| PR-010 | 2025-03-18 | Room 102 | 4 h | Bed rail | 0 | Confluent lysis |
| PR-011 | 2025-03-18 | Room 102 | 4 h | Monitor | 0 | Confluent lysis |
| PR-012 | 2025-03-18 | Room 102 | 4 h | Infusion pump | 0 | Confluent lysis |
| PR-013 | 2025-04-02 | Room 103 | 1 h | Bed rail | 0 | Confluent lysis |
| PR-014 | 2025-04-02 | Room 103 | 1 h | Stethoscope | 0 | Confluent lysis |
| PR-015 | 2025-04-02 | Room 103 | 1 h | Sink basin | 0 | Confluent lysis |
| PR-016 | 2025-04-02 | Room 103 | 4 h | Bed rail | 0 | Confluent lysis |
| PR-017 | 2025-04-02 | Room 103 | 4 h | Stethoscope | 0 | Confluent lysis |
| PR-018 | 2025-04-02 | Room 103 | 4 h | Sink basin | 0 | Confluent lysis |
| PR-019 | 2025-04-15 | Room 101 | 1 h | Faucet | 0 | Confluent lysis |
| PR-020 | 2025-04-15 | Room 101 | 1 h | Bed rail | 0 | Confluent lysis |
| PR-021 | 2025-04-15 | Room 101 | 1 h | Monitor | 0 | Confluent lysis |
| PR-022 | 2025-04-15 | Room 101 | 4 h | Faucet | 0 | Confluent lysis |
| PR-023 | 2025-04-15 | Room 101 | 4 h | Bed rail | 0 | Confluent lysis |
| PR-024 | 2025-04-15 | Room 101 | 4 h | Monitor | 0 | Confluent lysis |
| PR-025 | 2025-05-06 | Room 102 | 1 h | Door handle | 0 | Confluent lysis |
| PR-026 | 2025-05-06 | Room 102 | 1 h | Infusion pump | 0 | Confluent lysis |
| PR-027 | 2025-05-06 | Room 102 | 1 h | Bed rail | 0 | Confluent lysis |
| PR-028 | 2025-05-06 | Room 102 | 4 h | Door handle | 0 | Confluent lysis |
| PR-029 | 2025-05-06 | Room 102 | 4 h | Infusion pump | 0 | Confluent lysis |
| PR-030 | 2025-05-06 | Room 102 | 4 h | Bed rail | 0 | Confluent lysis |
| PR-031 | 2025-06-12 | Room 103 | 1 h | Monitor | 0 | Confluent lysis |
| PR-032 | 2025-06-12 | Room 103 | 1 h | Stethoscope | 0 | Confluent lysis |
| PR-033 | 2025-06-12 | Room 103 | 1 h | Sink basin | 0 | Confluent lysis |
| PR-034 | 2025-06-12 | Room 103 | 4 h | Monitor | 0 | Confluent lysis |
| PR-035 | 2025-06-12 | Room 103 | 4 h | Stethoscope | 0 | Confluent lysis |
| PR-036 | 2025-06-12 | Room 103 | 4 h | Sink basin | 0 | Confluent lysis |

**Notes:**

All samples were collected during the intervention phase (P2) of the study (January–August 2025).

PFU = plaque-forming units. Positive control: each batch of samples included a swab spiked with 10³ PFU of Ph‑kp8 to confirm assay sensitivity; all positive controls showed confluent lysis.

No PFU was detected in any of the 36 post‑intervention samples.

Surface types were selected from high‑touch areas as described in Methods section 2.7 and 2.8.
